# Supplementary material for: Pyruvate Dehydrogenase Kinase 1 inhibition mediated oxidative phosphorylation enhancement in cartilage promotes osteoarthritis progression
Source: BMC Musculoskelet Disord. 2023 Jul 20;24:597. doi: 10.1186/s12891-023-06585-6 (PMC10357736; doi:10.1186/s12891-023-06585-6)
Supplement: Supplementary file 1 — Additional file 1. [file 12891_2023_6585_MOESM1_ESM.zip › KEGG.Gsea.1653622432464/KEGG_LONG_TERM_DEPRESSION.html]

Details for gene set KEGG\_LONG\_TERM\_DEPRESSION[GSEA]

|  || Dataset | 55457\_collapsed\_to\_symbols.cls.cls #OA\_versus\_normal.cls.cls #OA\_versus\_normal\_repos |
| Phenotype | cls.cls#OA\_versus\_normal\_repos |
| Upregulated in class | normal |
| GeneSet | KEGG\_LONG\_TERM\_DEPRESSION |
| Enrichment Score (ES) | -0.23902127 |
| Normalized Enrichment Score (NES) | -0.95972496 |
| Nominal p-value | 0.5305466 |
| FDR q-value | 0.7363578 |
| FWER p-Value | 1.0 |
Table: GSEA Results Summary

  

Fig 1: Enrichment plot: KEGG\_LONG\_TERM\_DEPRESSION      
 Profile of the Running ES Score & Positions of GeneSet Members on the Rank Ordered List

  

| SYMBOL | TITLE | RANK IN GENE LIST | RANK METRIC SCORE | RUNNING ES | CORE ENRICHMENT || 1 | PLA2G12A | phospholipase A2 group XIIA [Source:HGNC Symbol;Acc:HGNC:18554] | 600 | 0.623 | -0.0084 | No |
| 2 | MAPK3 | mitogen-activated protein kinase 3 [Source:HGNC Symbol;Acc:HGNC:6877] | 757 | 0.579 | 0.0152 | No |
| 3 | RYR1 | ryanodine receptor 1 [Source:HGNC Symbol;Acc:HGNC:10483] | 1753 | 0.391 | -0.0382 | No |
| 4 | PLCB4 | phospholipase C beta 4 [Source:HGNC Symbol;Acc:HGNC:9059] | 2217 | 0.337 | -0.0535 | No |
| 5 | IGF1 | insulin like growth factor 1 [Source:HGNC Symbol;Acc:HGNC:5464] | 2286 | 0.328 | -0.0386 | No |
| 6 | GRIA2 | glutamate ionotropic receptor AMPA type subunit 2 [Source:HGNC Symbol;Acc:HGNC:4572] | 2342 | 0.322 | -0.0230 | No |
| 7 | PLA2G4A | phospholipase A2 group IVA [Source:HGNC Symbol;Acc:HGNC:9035] | 2656 | 0.284 | -0.0299 | No |
| 8 | GRM1 | glutamate metabotropic receptor 1 [Source:HGNC Symbol;Acc:HGNC:4593] | 3313 | 0.220 | -0.0674 | No |
| 9 | PLA2G10 | phospholipase A2 group X [Source:HGNC Symbol;Acc:HGNC:9029] | 3315 | 0.220 | -0.0540 | No |
| 10 | PLA2G3 | phospholipase A2 group III [Source:HGNC Symbol;Acc:HGNC:17934] | 3648 | 0.190 | -0.0681 | No |
| 11 | GNAI3 | G protein subunit alpha i3 [Source:HGNC Symbol;Acc:HGNC:4387] | 3694 | 0.186 | -0.0601 | No |
| 12 | PLCB1 | phospholipase C beta 1 [Source:HGNC Symbol;Acc:HGNC:15917] | 3724 | 0.183 | -0.0511 | No |
| 13 | PRKCA | protein kinase C alpha [Source:HGNC Symbol;Acc:HGNC:9393] | 3728 | 0.183 | -0.0400 | No |
| 14 | GUCY1B1 | guanylate cyclase 1 soluble subunit beta 1 [Source:HGNC Symbol;Acc:HGNC:4687] | 3962 | 0.159 | -0.0484 | No |
| 15 | PPP2CA | protein phosphatase 2 catalytic subunit alpha [Source:HGNC Symbol;Acc:HGNC:9299] | 4146 | 0.143 | -0.0539 | No |
| 16 | ITPR2 | "inositol 1,4,5-trisphosphate receptor type 2 [Source:HGNC Symbol;Acc:HGNC:6181]" | 4222 | 0.135 | -0.0514 | No |
| 17 | NOS1 | nitric oxide synthase 1 [Source:HGNC Symbol;Acc:HGNC:7872] | 4228 | 0.134 | -0.0435 | No |
| 18 | GRM5 | glutamate metabotropic receptor 5 [Source:HGNC Symbol;Acc:HGNC:4597] | 4941 | 0.077 | -0.0942 | No |
| 19 | PLA2G2D | phospholipase A2 group IID [Source:HGNC Symbol;Acc:HGNC:9033] | 5056 | 0.069 | -0.0989 | No |
| 20 | PPP1R17 | protein phosphatase 1 regulatory subunit 17 [Source:HGNC Symbol;Acc:HGNC:16973] | 5786 | 0.009 | -0.1551 | No |
| 21 | RAF1 | "Raf-1 proto-oncogene, serine/threonine kinase [Source:HGNC Symbol;Acc:HGNC:9829]" | 6187 | -0.019 | -0.1851 | No |
| 22 | CRHR1 | corticotropin releasing hormone receptor 1 [Source:HGNC Symbol;Acc:HGNC:2357] | 6390 | -0.034 | -0.1987 | No |
| 23 | PRKCB | protein kinase C beta [Source:HGNC Symbol;Acc:HGNC:9395] | 6453 | -0.039 | -0.2011 | No |
| 24 | GRIA3 | glutamate ionotropic receptor AMPA type subunit 3 [Source:HGNC Symbol;Acc:HGNC:4573] | 6454 | -0.039 | -0.1988 | No |
| 25 | LYN | "LYN proto-oncogene, Src family tyrosine kinase [Source:HGNC Symbol;Acc:HGNC:6735]" | 6514 | -0.043 | -0.2007 | No |
| 26 | GNAO1 | G protein subunit alpha o1 [Source:HGNC Symbol;Acc:HGNC:4389] | 6631 | -0.052 | -0.2065 | No |
| 27 | GUCY1A2 | guanylate cyclase 1 soluble subunit alpha 2 [Source:HGNC Symbol;Acc:HGNC:4684] | 6731 | -0.058 | -0.2107 | No |
| 28 | ITPR1 | "inositol 1,4,5-trisphosphate receptor type 1 [Source:HGNC Symbol;Acc:HGNC:6180]" | 6752 | -0.059 | -0.2085 | No |
| 29 | PLA2G5 | phospholipase A2 group V [Source:HGNC Symbol;Acc:HGNC:9038] | 6772 | -0.061 | -0.2063 | No |
| 30 | MAPK1 | mitogen-activated protein kinase 1 [Source:HGNC Symbol;Acc:HGNC:6871] | 6853 | -0.068 | -0.2083 | No |
| 31 | PLCB2 | phospholipase C beta 2 [Source:HGNC Symbol;Acc:HGNC:9055] | 7052 | -0.084 | -0.2186 | No |
| 32 | PLCB3 | phospholipase C beta 3 [Source:HGNC Symbol;Acc:HGNC:9056] | 7118 | -0.090 | -0.2181 | No |
| 33 | PPP2CB | protein phosphatase 2 catalytic subunit beta [Source:HGNC Symbol;Acc:HGNC:9300] | 7265 | -0.100 | -0.2233 | No |
| 34 | GNA12 | G protein subunit alpha 12 [Source:HGNC Symbol;Acc:HGNC:4380] | 7294 | -0.102 | -0.2192 | No |
| 35 | NRAS | "NRAS proto-oncogene, GTPase [Source:HGNC Symbol;Acc:HGNC:7989]" | 7300 | -0.102 | -0.2133 | No |
| 36 | PLA2G2E | phospholipase A2 group IIE [Source:HGNC Symbol;Acc:HGNC:13414] | 7486 | -0.117 | -0.2205 | No |
| 37 | GNAQ | G protein subunit alpha q [Source:HGNC Symbol;Acc:HGNC:4390] | 7518 | -0.120 | -0.2155 | No |
| 38 | CACNA1A | calcium voltage-gated channel subunit alpha1 A [Source:HGNC Symbol;Acc:HGNC:1388] | 7774 | -0.140 | -0.2268 | Yes |
| 39 | GNAZ | G protein subunit alpha z [Source:HGNC Symbol;Acc:HGNC:4395] | 7805 | -0.142 | -0.2204 | Yes |
| 40 | HRAS | "HRas proto-oncogene, GTPase [Source:HGNC Symbol;Acc:HGNC:5173]" | 7977 | -0.153 | -0.2243 | Yes |
| 41 | ARAF | "A-Raf proto-oncogene, serine/threonine kinase [Source:HGNC Symbol;Acc:HGNC:646]" | 8092 | -0.163 | -0.2231 | Yes |
| 42 | GNAI1 | G protein subunit alpha i1 [Source:HGNC Symbol;Acc:HGNC:4384] | 8110 | -0.164 | -0.2144 | Yes |
| 43 | BRAF | "B-Raf proto-oncogene, serine/threonine kinase [Source:HGNC Symbol;Acc:HGNC:1097]" | 8171 | -0.169 | -0.2086 | Yes |
| 44 | PLA2G1B | phospholipase A2 group IB [Source:HGNC Symbol;Acc:HGNC:9030] | 8232 | -0.173 | -0.2026 | Yes |
| 45 | CRH | corticotropin releasing hormone [Source:HGNC Symbol;Acc:HGNC:2355] | 8256 | -0.176 | -0.1936 | Yes |
| 46 | PRKCG | protein kinase C gamma [Source:HGNC Symbol;Acc:HGNC:9402] | 8582 | -0.200 | -0.2066 | Yes |
| 47 | GNAI2 | G protein subunit alpha i2 [Source:HGNC Symbol;Acc:HGNC:4385] | 8686 | -0.208 | -0.2018 | Yes |
| 48 | GNAS | GNAS complex locus [Source:HGNC Symbol;Acc:HGNC:4392] | 8929 | -0.226 | -0.2067 | Yes |
| 49 | GRID2 | glutamate ionotropic receptor delta type subunit 2 [Source:HGNC Symbol;Acc:HGNC:4576] | 8974 | -0.230 | -0.1959 | Yes |
| 50 | PRKG2 | protein kinase cGMP-dependent 2 [Source:HGNC Symbol;Acc:HGNC:9416] | 9126 | -0.242 | -0.1928 | Yes |
| 51 | GUCY1A1 | guanylate cyclase 1 soluble subunit alpha 1 [Source:HGNC Symbol;Acc:HGNC:4685] | 9420 | -0.266 | -0.1992 | Yes |
| 52 | MAP2K2 | mitogen-activated protein kinase kinase 2 [Source:HGNC Symbol;Acc:HGNC:6842] | 9932 | -0.315 | -0.2196 | Yes |
| 53 | PLA2G2A | phospholipase A2 group IIA [Source:HGNC Symbol;Acc:HGNC:9031] | 10152 | -0.336 | -0.2159 | Yes |
| 54 | MAP2K1 | mitogen-activated protein kinase kinase 1 [Source:HGNC Symbol;Acc:HGNC:6840] | 10197 | -0.340 | -0.1984 | Yes |
| 55 | ITPR3 | "inositol 1,4,5-trisphosphate receptor type 3 [Source:HGNC Symbol;Acc:HGNC:6182]" | 10390 | -0.360 | -0.1911 | Yes |
| 56 | PPP2R1A | protein phosphatase 2 scaffold subunit Aalpha [Source:HGNC Symbol;Acc:HGNC:9302] | 10395 | -0.360 | -0.1692 | Yes |
| 57 | KRAS | "KRAS proto-oncogene, GTPase [Source:HGNC Symbol;Acc:HGNC:6407]" | 10544 | -0.377 | -0.1575 | Yes |
| 58 | PPP2R1B | protein phosphatase 2 scaffold subunit Abeta [Source:HGNC Symbol;Acc:HGNC:9303] | 10578 | -0.382 | -0.1366 | Yes |
| 59 | PLA2G2F | phospholipase A2 group IIF [Source:HGNC Symbol;Acc:HGNC:30040] | 10628 | -0.386 | -0.1166 | Yes |
| 60 | PRKG1 | protein kinase cGMP-dependent 1 [Source:HGNC Symbol;Acc:HGNC:9414] | 10776 | -0.403 | -0.1032 | Yes |
| 61 | PLA2G6 | phospholipase A2 group VI [Source:HGNC Symbol;Acc:HGNC:9039] | 11128 | -0.446 | -0.1030 | Yes |
| 62 | JMJD7-PLA2G4B | JMJD7-PLA2G4B readthrough [Source:HGNC Symbol;Acc:HGNC:34449] | 12076 | -0.614 | -0.1390 | Yes |
| 63 | GRIA1 | glutamate ionotropic receptor AMPA type subunit 1 [Source:HGNC Symbol;Acc:HGNC:4571] | 12149 | -0.631 | -0.1057 | Yes |
| 64 | GNA11 | G protein subunit alpha 11 [Source:HGNC Symbol;Acc:HGNC:4379] | 12375 | -0.700 | -0.0800 | Yes |
| 65 | IGF1R | insulin like growth factor 1 receptor [Source:HGNC Symbol;Acc:HGNC:5465] | 12689 | -0.855 | -0.0517 | Yes |
| 66 | GNA13 | G protein subunit alpha 13 [Source:HGNC Symbol;Acc:HGNC:4381] | 12862 | -1.103 | 0.0030 | Yes |
Table: GSEA details [plain text format]

  

Fig 2: KEGG\_LONG\_TERM\_DEPRESSION      
 Blue-Pink O' Gram in the Space of the Analyzed GeneSet

  

Fig 3: KEGG\_LONG\_TERM\_DEPRESSION: Random ES distribution      
 Gene set null distribution of ES for **KEGG\_LONG\_TERM\_DEPRESSION**
